# Presence of Phylloquinone in the Intraerythrocytic Stages of *Plasmodium falciparum*

**DOI:** 10.3389/fcimb.2022.869085

**Published:** 2022-04-21

**Authors:** Rodrigo A. C. Sussmann, Heloisa B. Gabriel, Alejandro García Ríos, Danielle S. Menchaca Vega, Lydia F. Yamaguchi, Antonio Doménech-Carbó, Gerardo Cebrián-Torrejón, Emilia A. Kimura, Massuo J. Kato, Ignasi Bofill Verdaguer, Marcell Crispim, Alejandro M. Katzin

**Affiliations:** ^1^ Department of Parasitology, Institute of Biomedical Sciences, University of São Paulo, São Paulo, Brazil; ^2^ Center for Environmental Sciences, Institute of Humanities, Arts and Sciences, Federal University of Southern Bahia, Porto Seguro, Brazil; ^3^ Laboratory of Environmental Chemistry and Metalopharmaceuticals, Institute of Chemistry at the University of São Paulo, São Paulo, Brazil; ^4^ Chemistry Program, Universidad del Quindio, Quindio, Colombia; ^5^ Department of Fundamental Chemistry, Institute of Chemistry, University of São Paulo, São Paulo, Brazil; ^6^ Departament of Analytic Chemistry, Facultat de Química, Universitat de València, Valencia, Spain; ^7^ Laboratoire Connaissance et Valorisation Equipes d'Accueil (COVACHIM-M2E EA) 3592, Université des Antilles, Pointe-à-Pitre Cedex, Pointe-à-Pitre, Guadeloupe, France

**Keywords:** malaria, plasmodium, isoprenoid, phylloquinone, phytol, phytyl, naphthoquinone, atovaquone

## Abstract

Malaria is one of the most widespread parasitic diseases, especially in Africa, Southeast Asia and South America. One of the greatest problems for control of the disease is the emergence of drug resistance, which leads to a need for the development of new antimalarial compounds. The biosynthesis of isoprenoids has been investigated as part of a strategy to identify new targets to obtain new antimalarial drugs. Several isoprenoid quinones, including menaquinone-4 (MK-4/vitamin K2), α- and γ-tocopherol and ubiquinone (UQ) homologs UQ-8 and UQ-9, were previously detected in *in vitro* cultures of *Plasmodium falciparum* in asexual stages. Herein, we described for the first time the presence of phylloquinone (PK/vitamin K1) in *P. falciparum* and discuss the possible origins of this prenylquinone. While our results in metabolic labeling experiments suggest a biosynthesis of PK prenylation *via* phytyl pyrophosphate (phytyl-PP) with phytol being phosphorylated, on the other hand, exogenous PK attenuated atovaquone effects on parasitic growth and respiration, showing that this metabolite can be transported from extracellular environment and that the mitochondrial electron transport system (ETS) of *P. falciparum* is capable to interact with PK. Although the natural role and origin of PK remains elusive, this work highlights the PK importance in plasmodial metabolism and future studies will be important to elucidate in seeking new targets for antimalarial drugs.

## Introduction

Malaria is one of the most widespread parasitic diseases, with a high social and economic impact that contributes to poverty in Africa, Southeast Asia and South America (World Health Organization, 2021). In 2020, malaria affected 241 million people and resulted in 627,000 deaths, most of them in Africa, due to *Plasmodium falciparum* infections (World Health Organization, 2021). One of the greatest problems for controlling the disease is the emergence of drug-resistant strains, which demands efforts to discover new antimalarial compounds (World Health Organization, 2021).

One leading drug development strategy is to target plant-like pathways of the parasites involved in the formation of essential compounds. The presence of these pathways results from an endosymbiotic event between a red algae and the apicomplexan phagotrophic ancestor (Janouskovec et al., 2010). Due to this phenomenon, *P. falciparum* possess a non-photosynthetic plastid called apicoplast, which physically and metabolically interacts with mitochondria during the asexual life cycle (van Dooren et al., 2005; Seeber and Soldati-Favre, 2010).

The most studied plant-like biosynthesis pathways in parasites include the shikimate and methylerythritol 4-phosphate (MEP) pathways, which are responsible for chorismate and isoprenoid biosynthesis, respectively (Keeling et al., 1999; Cassera et al., 2004; Seeber and Soldati-Favre, 2010). The MEP pathway provides the basic isoprene unit of five carbons called isopentenyl pyrophosphate (IPP) and its isomer dimethylallyl pyrophosphate (DMAPP). IPP and DMAPP are the simplest isoprene units used by prenyltransferases/synthases to build up all meroterpenes, polyisoprenoids and higher terpenes (Lange et al., 2000). All living organisms produce isoprene chain-based compounds such as geranyl pyrophosphate (GPP, 10 carbons), farnesyl pyrophosphate (FPP, 15 carbons) and geranylgeranyl pyrophosphate (GGPP, 20 carbons) (Ogura et al., 1997). Moreover, compounds with C-20 partially saturated isoprene chains (also known as phytyl pyrophosphate, phytyl-PP) are found in photosynthetic prokaryotes, archaea, and plants (Ischebeck et al., 2006; Sasaki et al., 2011; Meadows et al., 2018). Partially or fully saturated 20-carbon polyisoprenoids can be formed by the hydrogenation of the geranylgeranyl chain attached to chlorophyll intermediates in photosynthetic organisms or directly from geranylgeraniol/GGPP in prokaryotes, archaea and plants (Sasaki et al., 2011; Meadows et al., 2018). This last process is catalyzed by geranylgeranyl reductases (GGRs) dependent on NADH or FADH (Sasaki et al., 2011; Meadows et al., 2018). Moreover, the ability of plants to “recycle” phytol from chlorophyll degradation through its phosphorylation has been described (Ischebeck et al., 2006). Specifically, phytol is phosphorylated to phytyl-phosphate and phytyl- pyrophosphate by the action of kinases dependent on cytidine triphosphate (CTP), guanidine triphosphate (GTP), uridine triphosphate (UTP) or adenine nucleoside triphosphate (ATP) (Ischebeck et al., 2006).

The presence of α- and γ-tocopherol (vitamin E), phytylated isoprenoid quinones, in *P. falciparum* was previously discovered by our group (Sussmann et al., 2011), as well as the biosynthesis of other isoprenoid quinones such as UQ-8 and 9 (Ellis, 1994) and menaquinone-4 (vitamin K2, MK-4) (Tonhosolo et al., 2010). Tocopherol can be part of the parasitic antioxidant defense since its biosynthesis is stimulated under high oxygen saturation (Sussmann et al., 2017). Regarding vitamin K2, it was demonstrated that mitochondrial NADH dehydrogenase from *P. falciparum* can use MK-4/menadione to oxidize NADH (Dong et al., 2009; Tonhosolo et al., 2010), and the ubiquinone (UQ)/MK-4 pool ratio was reversed when a parasite culture was maintained under microaerophilic conditions, suggesting that MK-4 has a role in the mitochondrial electron transport system (ETS) (Tonhosolo et al., 2010).

Since the biosynthesis of tocopherol and MK-4 has been described, it was suggested that the biosynthetic pathway for phytyl-PP and the naphthoquinone ring that composes phylloquinone (2-methyl-3-phytyl-1,4-naphthoquinone/PK/vitamin K1) could also be active (Nowicka and Kruk, 2010). Briefly, the classic route for PK biosynthesis in plants requires chorismate, which is provided by the shikimate pathway and then converted to isochorismate by the isochorismate synthase enzyme (MenF, EC number: 5.4.4.2). Isochorismate undergoes six modifications of its aromatic head group up to the first specific metabolite of PK biosynthesis: 2-carboxy-1,4-naphthoquinone, which is phytylated by the enzyme 1,4-dihydroxy-2-naphthoate polyprenyltransferase (MenA; EC number: 2.5.1.130). Finally, a *S*-adenosyl-*L*-methionine-dependent methylation is carried out by the enzyme 2-methoxy-6-polyprenyl-1,4-benzoquinol methylase (MenG; EC number: 2.1.1.329).

As part of the group of vitamin K, along with MK-4 and menadione (Nowicka and Kruk, 2010), the well-known function of PK in plants is to act as an electron carrier for oxygenic photosynthesis coupled to photosystem I (PSI) and oxidative folding of photosystem II subunits (Basset et al., 2017). However, recent findings demonstrated an alternative targeting of phylloquinone for transmembrane redox signaling associated with parasitism in nonphotosynthetic holoparasites plants (Xi et al., 2021). The presence of PK in plant membranes suggests the involvement of PK-mediated ETS acting as a redox mediator (Lochner et al., 2003; Xi et al., 2021). Herein, we described the identification of PK in the intraerythrocytic stages of *P. falciparum* and discuss about the probable functions and origins of this molecule in the parasite.

## Materials and Methods

### Reagents

Albumax I and RPMI-1640 were purchased from Fisher Scientific^®^ (Leicestershire, UK). All solvents used were HPLC-grade or higher quality and purchased from Sigma-Aldrich (St. Louis, Missouri USA). Radiolabeled isoprenic precursors [1-(n)-^3^H] geranylgeranyl pyrophosphate triammonium salt {[1-(n)-^3^H]-GGPP; 14 Ci/mmol}, [1-(n)-^3^H] phytol (20 Ci/mmol), and {[1-(n)-^3^H] phytyl-PP ([^3^H]-phytyl-PP; 20 Ci/mmol) were obtained from Amersham-Pharmacia Biotech (Buckinghamshire, UK). Adenosyl-*L*-methionine, S-[methyl-^3^H] ([^3^H]-SAM, 82 Ci/mmol) and [1-(n)-^3^H] FPP triammonium salt ([^3^H]-FPP 23 Ci/mmol) were purchased from Perkin Elmer^®^ (Waltham, Massachusetts, EUA). PK, α-tocopherol, γ-tocopherol, phytol, phytyl-PP, GGPP, UQ-8, UQ-9 and MK-4 pure standards were purchased from Sigma-Aldrich. Saponin, hypoxanthine, gentamycin sulfate, D-sorbitol, glucose, 4-(2-hydroxyethyl)-1-piperazineethanesulfonic acid (HEPES) and another reagent not cited here were also purchased from Sigma-Aldrich.

### P. falciparum In Vitro Culture

*P. falciparum* clone 3D7 was cultured *in vitro* by the classic culture methodology reported by Trager and Jensen (Trager and Jensen, 1976) employing RPMI-1640 medium completed supplemented with Albumax I (0.5%) in 75 cm^3^ cell culture flasks (Radfar et al., 2009). The pH was adjusted to 7.4, and a gas mixture of 5% CO_2_, 5% O_2_, and 90% N_2_ was employed. Culture synchronization at ring stages was performed with 5% (w/v) D-sorbitol solution as described by Lambros & Vanderberg (Lambros and Vanderberg, 1979). Parasite development was monitored by Giemsa-stained thin smear microscopy. Blood was leuco-depleted, and PCR (for mycoplasma) and optic microscopy were used to monitor culture contamination (Rowe et al., 1998).

### Metabolic Labeling

Synchronous cultures of *P. falciparum* were labeled at ring stages with 15 μCi/ml [^3^H]-GGPP, [^3^H]-FPP, [^3^H]-phytyl-PP or [^3^H]-phytol or 2.5 μCi/ml [^3^H]-SAM in complete RPMI 1640 medium for 16-18 h. After the labeling period, infected erythrocytes containing late trophozoite or schizont stages were isolated by magnetic separation (see below) or purified discontinuous Percoll^®^ (see below). For all metabolic labeling experiments, approximately 4x10^8^ infected erythrocytes were used.

### Isolation of Rings, Trophozoites and Schizonts From Infected Erythrocytes

Cultures at the schizont stage were purified by a magnetic column (MACS separation column, Manual Cell Separation) (Ribaut et al., 2008). Briefly, the column was equilibrated and washed at room temperature with RPMI-1640 complete medium. The culture was centrifuged, and the pellet was suspended in complete culture medium (1:10 v/v) and loaded into the column. Then, the column was washed with RPMI-1640 complete medium, and the retained parasites were collected (Ribaut et al., 2008). Infected red blood cells were centrifuged at 600 x *g* for 5 min, and the pellet was frozen in liquid nitrogen for further analysis.

Ring (1-20 h after reinvasion), trophozoite (20-30 h after reinvasion) and schizont (30-45 h after reinvasion) forms were purified with a 40/70/80% discontinuous Percoll^®^ gradient (10,000 x *g*, 30 min, 25°C) (Braun-Breton et al., 1986). In the fraction containing the ring stage and uninfected erythrocytes (80%), parasites were isolated by treatment with 0.1% (w/v) saponin for 5 min followed by two washes with PBS, pH 7.2, at 10,000 x *g* for 10 min. The fractions containing trophozoite stages or schizont stages were centrifuged at 600 x *g* for 5 min, and the pellets were frozen in liquid nitrogen for further analysis.

### Isolation of Free Parasites

The parasitophorous vacuole/erythrocyte-free parasites for phytol phosphorylation assays were obtained by saponin lysis (Christophers and Fulton, 1939). For this purpose, erythrocytes were suspended in 30 mL of PBS (30 mM Na_2_HPO_4_, 6 mM KH_2_PO_4_, pH 7.4, 120 mM NaCl) with 2 g/L glucose and 0.2% (v/v) saponin at 4°C and centrifuged at 700 x *g* at 4°C. The resulting pellet was washed twice in 30 ml of complete cold RPMI-1640 medium. Finally, the pellet was frozen in liquid nitrogen for posterior analysis.

### PK Extraction

Lyophilized infected erythrocytes (1 x 10^10^) were suspended in 4 mL of deionized water in glass tubes. Cell lysis was carried out at 4°C by ultrasonication with 4 pulses of 5 seconds with 10% intensity and 15 second intervals between them. The proteins were precipitated by adding 800 µL of ethanol. After mixing for 1 min, extraction was performed three times with 8 mL of hexane. The sample was shaken for 1 min and centrifuged at 2700 x *g* for 10 min at 4°C (Ischebeck et al., 2006). The hexane extract of erythrocytes infected with *P. falciparum* was concentrated under nitrogen flow and analyzed by RP-HPLC.

### Phytyl-PP Extraction

The lysis of 1 x 10^10^ erythrocytes infected with *P. falciparum* was achieved by sonication as described for PK and phytol extraction. The extraction of phytyl-PP was carried out three times with 6 mL of n-butyl alcohol saturated with water. The solution was centrifuged at 3000 x *g* for 10 min at 4°C. The n-butyl alcohol phases were combined and evaporated under nitrogen stream and analyzed by TLC.

The samples were dissolved in a small volume of n-butyl alcohol saturated with water and applied on a silica 250-PA TLC plate (Mallinckrodt Baker, Griesheim, Germany). The plate was developed with isopropyl alcohol/ammonia (32%)/H_2_O (6:3:1 v/v/v). Phytyl-PP was identified by retention factor (R*_f_*) and coelution with a commercial standard (Ischebeck et al., 2006).

### Phytol Phosphorylation Assay

The methodology for measuring phytol phosphorylation activity in parasite extracts was carried out as previously reported (Ischebeck et al., 2006). Approximately 4x10^9^ saponin-purified free schizonts were resuspended in a solution containing 100 mM Tris-HCl buffer, pH 7.5, 1 mM dithiothreitol (DDT); 1 mM MgCl_2_; 1 mM isoascorbate; 1 mM KCl; 0.1% albumin serum; and a protease inhibitor cocktail purchased from Sigma. Then, parasites were successively frozen and thawed 4 times in liquid nitrogen. Subsequently, parasites were lysed three times by 10 second sonications at 30 second intervals. Then, samples were centrifuged at 100,000 x *g* for 30 min at 4°C (Ischebeck et al., 2006), yielding a membrane fraction (pellet) and a cytoplasmic fraction (supernatant).

Both phases were separated, and 300 μL of reaction buffer was added (50 mM MgCl_2_; 20 mM NaF; 50 mM Tris-HCl, pH 8; 20 mM sodium orthovanate; 1 mM DTT; 50 mM CaCl_2_; 100 pmol [^3^H]-phytol). Finally, ATP, CTP, UTP and GTP were added to a final concentration of 200 mM each. After 30 min incubation at 37°C, the reaction was stopped by adding 300 μl of saturated n-butanol (1:1 v/v). The sample was then vortexed and centrifuged at 10,000 x *g* for 10 min. The supernatant was collected, and the extraction procedure was repeated twice again by adding the same volume of saturated butanol (1:1 v/v). The three collected supernatants were transferred to a single glass tube and dried under a nitrogen stream. For chromatographic analysis, the sample was suspended on the RP-HPLC initial mobile phase (system II, see below) using phytol, phytyl-P and phytyl-PP as internal standards (Ischebeck et al., 2006).

### RP-HPLC Analysis

The stationary phase was a Phenomenex Luna^®^ C18 column (250 mm x 4.6 mm x 5 μm) (Phenomenex^®^, CA, USA) coupled to a pre-C18 column (Phenomenex^®^, CA, USA). A UV-vis or a 170 diode array detector (DAD) (Gilson^®^, Villiers-le Bel, France) and an FC203B fraction collector were used. The software used for data processing was the Trilution™ LC 3.0 System Software. Three different elution systems were used to analyze the samples.

**System I:** A linear gradient with methanol (solvent A) and acetonitrile (solvent B) was used at a flow rate of 1 mL/min. The ratio was initially 50% (B), which increased to 70% (B) in 30 min and was then maintained for 20 min. The fractions were collected once per minute. Pure standards of UQ-8, UQ-9, MK-4 and PK were co-injected. As the intraerythrocytic stages of *P. falciparum* biosynthesize UQ-8, UQ-9 and MK-4, the pure standards of these compounds were injected in this system to show that they do not co-elute with PK and tocopherol. UQs were monitored at 290 nm, MK-4 at 270 nm and PK at 245 nm.

**System II:** The system consisted of a linear gradient elution with 25 mM NaHCO_3_ (A) and acetonitrile (B). The gradient started at 30% (B) and increased to 0% (B) in 20 min, was maintained for 19 min and returned to initial conditions in two minutes. The flow rate was set at 1 mL/min, the fractions were collected once per minute, and internal standards were monitored at 214 nm.

**System III:** An isocratic system with methanol/ethanol (1:1 v/v) was used at a flow rate of 0.5 mL/min. Samples were collected once per minute. The pure standards of UQ-8, UQ-9 and MK-4 were injected into this system to assess whether they co-eluted with PK and were monitored at the wavelengths described above.

### Gas Chromatography-Mass Spectrometry Analysis

An electron impact gas chromatography-mass spectrometer (GC-MS) with a Trace GC and Y2K ion trap PolarisQ MS System (Finnigan, ThermoQUEST Inc., San Jose, CA) with a data analysis program (Xcalibur version 1.3) was used. The device was equipped with a TR-1MS column (30 m, 0.25 mm, 0.25 μm, Thermo Scientific, USA, CA). The injector temperature was set at 220°C, and there was a splitless liner. An oven temperature of 100°C was initially maintained for 2 min and then increased to 300°C at a rate of 25°C/min. The temperature was maintained for 10 min and then reduced to its initial value. The transfer line was maintained at 260°C, and the helium flow was 1.5 mL/min. The MS was operated in positive mode with an ion source at 200°C. The mass range monitored was *m/z* 40 to 500 (full scan). Vitamin K1 and its derivatives were identified by the ions at m/z 450 for PK (Sussmann et al., 2016), m/z 452 for KH_2_ and *m/z* 480 for methylated hydrophylloquinone.

### LC/MS Analysis

**System I:** The analysis was performed in an LCQ Duo™ MS (ThermoFinnigan, EUA) coupled with Nano RP-HPLC (LC-Packings, model Ultimate) operated with a flux of 250 µL/min. The solvent was 80% acetonitrile with 0.2% lithium iodide. Electrospray ionization (ES) was used, and the MS was operated in positive mode with a potential of 4.5 kV and a capillary temperature of 180°C. The mass spectra were processed with Xcalibur^®^ 2.0 software (Copyright^©^ Thermo Electron Corporation, USA).

**System II:** The analysis was performed with a MicrOTOF-QII MS (Bruker^®^). The equipment was operated in negative mode with capillary voltage of 3500 V, N_2_ as a mixture gas at 2 bar, N_2_ as a drying gas at 4 L/min and a drying temperature of 180°C. Funnel 1 was an RF funnel with 400 Vpp, a quadrupole energy of 6 eV, collision energy of 12 eV and the hexapole RF was operated at 200 Vpp. In tandem MS (MS/MS), collision energies of 0 to 20 eV were used.

**System III:** The analysis was performed with a High-Resolution LC-QToF - maXis II™ (Bruker^®^). The column was a Shimadzu XR ODSIII (150x2 mm) with flux 0.2 ml/min. The solvent was 60% acetonitrile and 40% methanol at 40°C.

The equipment was operated with atmospheric pressure chemical ionization (APCI) in positive mode with capillary voltage of 3500 V, N_2_ as a mixture gas at 3 bar, N_2_ as a drying gas at 4 L/min and a drying temperature of 250°C. Funnel 1 was an RF funnel with 400 Vpp, a quadrupole energy of 7 eV, collision energy of 6 eV and the hexapole RF was operated at 700 Vpp.

Each extract obtained from infected erythrocytes, uninfected erythrocytes and standard were filtered and injected into the LC-QToF equipment. Prior to the injection of the samples, system suitability tests and blanks were carried out between each of them.

### Electrochemical Study

Electrochemical measurements were performed at 298 ± 1 K in a thermostatic cell with CH 660I equipment. A BAS MF2012 glassy carbon working electrode (geometrical area 0.071 cm^2^), a platinum wire auxiliary electrode and an Ag/AgCl (3 M NaCl) reference electrode were used in a conventional three-electrode arrangement. Cyclic and square wave voltammetry (CV and SWV, respectively) were used as detection modes. Eventually, derivative convolution of the data was performed to increase the peak resolution. Thin films of vitamin K1 and lyophilized blood samples were prepared on GCEs following a previously reported procedure (Doménech-Carbó et al., 2013b) by pipetting 10 µL of the liquid vitamin or 20 µL of a dispersion (1 mg/mL) of blood in ethanol and allowing the solvent to evaporate in air. As a result, a uniform, fine coating of the solid was adhered to the working electrode. Aqueous 0.10 M potassium PBS at physiological pH that had been previously degasified by bubbling argon for 10 min was used as a supporting electrolyte.

### Methylation of Hydrophylloquinone

The stabilization of hydrophylloquinone was achieved by di-*O*-methylation as described (Sussmann et al., 2016). Briefly, 4x10^8^ infected erythrocytes labeled with [^3^H]-phytyl-PP were lysed by ultrasonication in a Branson sonicator at 4°C with three pulses of 5 second duration with 10% potency and 10 second intervals between them. Then, 50 mg of potassium hydroxide and 60 μL of methyl iodide (CH_3_I) were added. The reaction was stirred overnight under a nitrogen atmosphere at room temperature in the dark. The reaction mixture was partitioned between water (2 mL) and dichloromethane (3 × 2 mL). The organic phase was evaporated under nitrogen stream, and the residue was submitted to RP-HPLC analysis and scintillation counting.

### Atovaquone Rescue Assays

For atovaquone (ATO) IC_50_ value estimation, parasites were incubated for 48 _h_ in the absence or presence of several concentrations of PK: (i) 0.010 µM was used as the first concentration of ATO, which reached 0.000156 µM by serial dilution (1:1/vol:vol), and (ii) 5.63 µM was used as the first concentration of PK, which reached 0.09 µM by serial dilution (1:1/vol:vol). For MK-4 IC_50_ value estimation, parasites were incubated for 48 _h_ in the absence or presence of MK-4; 16 µM was the first of several concentrations of MK-4 used.

Assays were performed in 96-well plates starting at 2% parasitemia in the ring stage and 2% hematocrit as described (Desjardins et al., 1979). Parasite growth was monitored after 48 h by SYBR Green I^®^ DNA staining (Smilkstein et al., 2004). For this purpose, 100 μL of culture was incubated in darkness and at room temperature after adding 100 μL of Syber Green I^®^ 2/10,000 (v/v) in lysis buffer [20 mM Tris, pH 7.5; 5 mM EDTA; 0.008% saponin (w/v); 0.08% Triton X-100 (v/v)]. After 1 h, fluorescence was measured with a POLARstar Omega fluorometer^®^ (BMG Labtech^®^, Ortenberg, Germany) with excitation and emission bands centered at wavelengths of 485 and 530 nm, respectively. Inhibition of parasite growth was analyzed in relation to the logarithm of the concentration using a nonlinear regression (dose-response slope/variable sigmoid equation) by using GraphPad Prism^®^ software. Only experiments showing R-squared values (R^2^) ≥ 0.95 were accepted. All experiments were performed at least three times with three technical triplicates for each experiment.

### Oxygen Consumption Assays

Oxygen consumption assays were performed in cultures at the trophozoite/young schizont stages (Murphy et al., 1997). Red blood cells were centrifuged at 1500 x *g* for 5 min at 4°C and lysed with 30 mL of 0.03% saponin in PBS at 25°C for 5 min. The obtained sample was centrifuged at 1500 x *g* for 5 min at 4°C and washed in respiration buffer (125 mM sucrose, 65 mM KCl, 10 mM HEPES-KOH, pH 7.2, 5 mM MgCl_2_, 2 mM KH_2_PO_4_, 0.5 mM EGTA). Free parasites were suspended to 1x10^9^ cells/ml in respiration buffer, and the samples were utilized immediately for oxygen consumption measurements with a high-resolution oxygraph (Oxygraph-2k Oroborus Instruments, Innsbruck, Austria). ATO, PK, carbonyl cyanide-4-(trifluoromethoxy) phenylhydrazone (FCCP) or antimycin A (AntA) additions were performed during the assay as described in the results.

## Results

### Detection of Phylloquinone During the Intraerythrocytic Stages of P. falciparum

**Figure 1:**
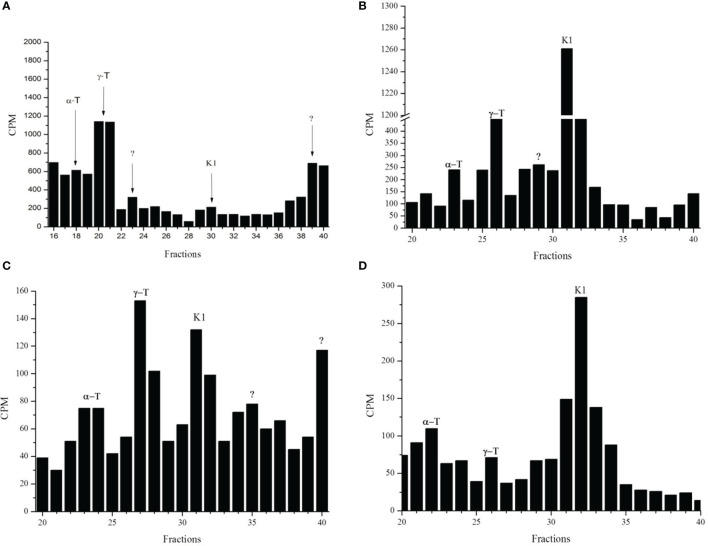
Radioactive incorporation profile for phytylated quinones in *P. falciparum* in schizont stage. Infected erythrocytes with schizont stages metabolically labeled with [^3^H]-phytol **(A)**, [^3^H]-GGPP **(B)**, [^3^H]-FPP **(C)** or [^3^H]-SAM **(D)** were subjected to hexane extraction. These extracts were separated by RP-HPLC (System I). Radioactive fractions were coincident with the corresponding standards γ-T (γ-tocopherol), α-T (α-tocopherol), K1 (phylloquinone), and “?” (not identified). The experiment was performed three times by using isoprene precursors and twice by using [^3^H]-SAM.

Radioactive fractions with retention times coincident with the standards α-tocopherol, γ-tocopherol and phylloquinone (PK) were observed in schizont stages of *P. falciparum* when [^3^H]-phytol (**Figure 1A**), [^3^H]-GGPP (**Figure 1B**), [^3^H]-FPP (**Figure 1C**), [^3^H]-SAM (**Figure 1D**), [^3^H]-phytyl-PP (**Figure 4**) were used as precursors and analyzed by reverse-phase high-performance liquid chromatography (RP-HPLC) using solvent system I. Using another RP-HPLC method (solvent system III) peaks with retention times coincident with the standards MK-4 and PK in the three stages of intraerythrocytic *P. falciparum* labeled with [^3^H]-GGPP were identified (supplemental data, **Figure S1A**). The greatest incorporation of the radioactive precursor was detected in the schizont stage. Similar results were found in the schizont stage when [^3^H]-FPP was used as a precursor and samples were analyzed by the system III RP-HPLC method (supplemental data, **Figure S1B**). The incorporation of [^3^H]-FPP and [^3^H]-GGPP into phytylated quinones suggests that parasites biosynthesize *de novo* phytyl-PP. Moreover, the use of [^3^H]-SAM shows that the probable PK and tocopherols were methylated as expected (Threlfall et al., 1968; Gaudillière et al., 1984).

### Phylloquinone Mass Spectrometry Analysis

**Figure 2:**
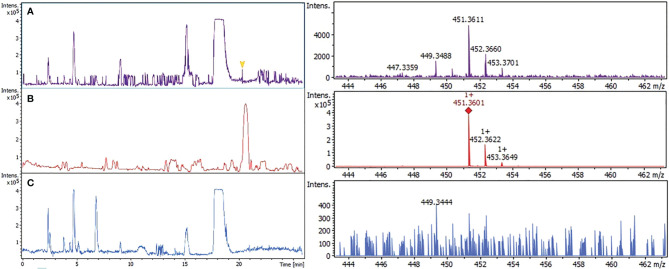
Confirmation of phylloquinone by high resolution mass spectrometry (LC-QToF*/*system III). Three different extracts were analyzed. Infected erythrocytes **(A)**; phylloquinone standard **(B)**; uninfected erythrocytes **(C)**. The signal obtained at 20.3 min of all fractions were compared with their mass spectrum.

The presence of PK in the parasite was confirmed by two methods of mass spectrometry. First, using liquid chromatographic separation and high-resolution mass spectrometry with atmospheric pressure chemical ionization LC-APCI-QToF. Chromatographic signal (**Figure 2A**) detected at 20.3 min in infected erythrocytes with molecular ion mass [M-H]^+^ of *m/z* 451.3611 Da was only 2.2 ppm different from the signal presented by phylloquinone standard, *m/z* 451.3601 Da (**Figure 2B**). Uninfected erythrocytes showed no signal (**Figure 2C**). Second, using the liquid chromatography/electrospray ionization mass spectrometry (LC/ESI) system I. The molecular ion peaks at *m/z* 456 and 439 Da corresponded to [M + Li] and [M + Li-H_2_O], respectively, considering the molecular formula C_31_H_46_O_2_ (supplemental data, **Figure S2**). The fragmentary ions at *m/z* 204, 221 and 245 Da were identified as the aromatic moieties of PK (supplemental data, **Figure S2A**). A similar profile was detected in infected erythrocytes (1 x 10^10^ parasite extracts) (supplemental data, **Figure S2B**) but with 3.5-fold higher intensity than the same amounts of uninfected erythrocytes (supplemental data, **Figure S2C**), comparing peak area and total ion count (TIC) for each sample.

The presence of PK was not detected in 100 mL of lyophilized complete culture medium extracted as described for the parasites (**Figure S2D**), suggesting that this vitamin is inherent to the complex erythrocyte-parasite. Although vitamin K1 is not biosynthesized by humans, when provided in the diet, it can be transported by plasma lipoproteins and found in the bloodstream (Shearer, 1992; Shearer et al., 2012).

### Electrochemical Study

The structure of PK determined by mass spectrometry was further analyzed by electrochemical studies to confirm its presence and the oxidation state of this compound in infected erythrocytes. The proposed methodology explores the electroactive character of both vitamin K1, which is dominated by a quinone/hydroquinone redox interconversion (Munir et al., 2012; Webster, 2012), and Fe-heme, where the Fe(III)/Fe(II) redox pair has been widely studied (Lexa et al., 1988; Chen et al., 1990; Tieman et al., 1990; Jeon and Bruice, 1992; Sagara et al., 1998; de Groot et al., 2005). The electrochemistry of vitamin K3 was studied by Zhu and Li (Zhu and Li, 1999). Here, the voltammetry of microparticles (VMP), a technique that enables the determination of the voltammetric response of sparingly soluble solid materials (Doménech-Carbó et al., 2013a), was applied to vitamin K1 films deposited on glassy carbon electrodes (GCEs) and microparticulate deposits of uninfected blood and infected erythrocytes following reported procedures (Doménech-Carbó et al., 2013b).

**Figure 3:**
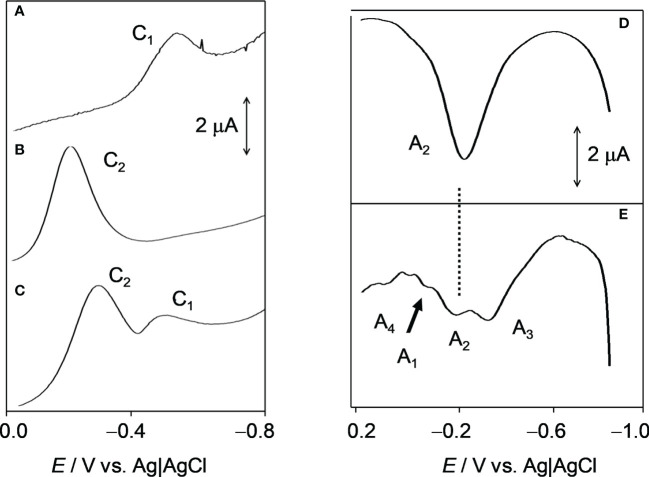
Square wave voltammetric curves of a vitamin K1 film on a glassy carbon electrode **(A)** and deposits of uninfected blood **(B)** and infected erythrocytes **(C)** on a glassy carbon electrode in contact with 0.10 M phosphate buffer saline at pH 7.4. Potential scan initiated at 0.0 V in the negative direction (potential step increment, 4 mV; square wave amplitude, 25 mV; frequency, 10 Hz). Square wave voltammetric curves of deposits of uninfected **(D)** and *P. falciparum-*infected erythrocytes **(E)** on glassy carbon electrode in contact with 0.10 M PBS at pH 7.4. Potential scan initiated at −0.85 V in the positive direction; potential step increment, 4 mV; square wave amplitude, 25 mV; frequency, 10 Hz. C_1_, C_2_, (A_1_, A_2_, A_3_, A_4_) denote cathodic (anodic) signals corresponding to electrochemical reduction (oxidation) processes described in detail in the text.

**Figure 3** compares the square wave voltammetry of vitamin K1, uninfected blood and infected erythrocytes in contact with phosphate-buffered saline (PBS) when the potential is scanned in the negative direction. Vitamin K1 displayed a unique cathodic signal (C_1_) at −0.52 V (**Figure 3A**), which can be assigned to the proton-assisted reduction of the quinone unit, whereas for uninfected blood, a reduction signal at −0.20 V (C_2_) was recorded (**Figure 3B**). This signal appears as a single, undifferentiated peak, which can be attributed to the reduction of Fe(III)-heme to Fe(II)-heme. Remarkably, the voltammograms for *P. falciparum*-infected erythrocytes showed both peaks C_1_ and C_2_ (**Figure 3C**), thus denoting the presence of PK accompanying Fe-heme to some extent forming a hemoglobin-phylloquinone complex (Zhu and Li, 1999).

When the potential is scanned in the positive direction, the vitamin K1 displays an anodic signal at 0.0 V (A_1_) corresponding to the anodic counterpart of the process C_1_. Similarly, the uninfected erythrocytes (**Figure 3D**) yielded peak A_2_ at -0.20 V, corresponding to the reversal of the process C_2_. Remarkably, the voltammogram of the infected erythrocytes (**Figure 3E**) showed overlapping peaks at -0.30 (A_3_), -0.20 (A_2_), 0.00 (A_1_) and +0.10 V (A_4_). Peak A_3_ can in principle be attributed to the oxidation of a Fe(II)-heme form differing from the pristine Fe(II)-heme produced in uninfected erythrocytes conceivably the farnesylated species generated by action of *P. falciparum*. However, the multiple anodic peak profile suggests that other reduced species were generated in the infected erythrocytes.

### Methylation of Hydrophylloquinone

**Figure 4:**
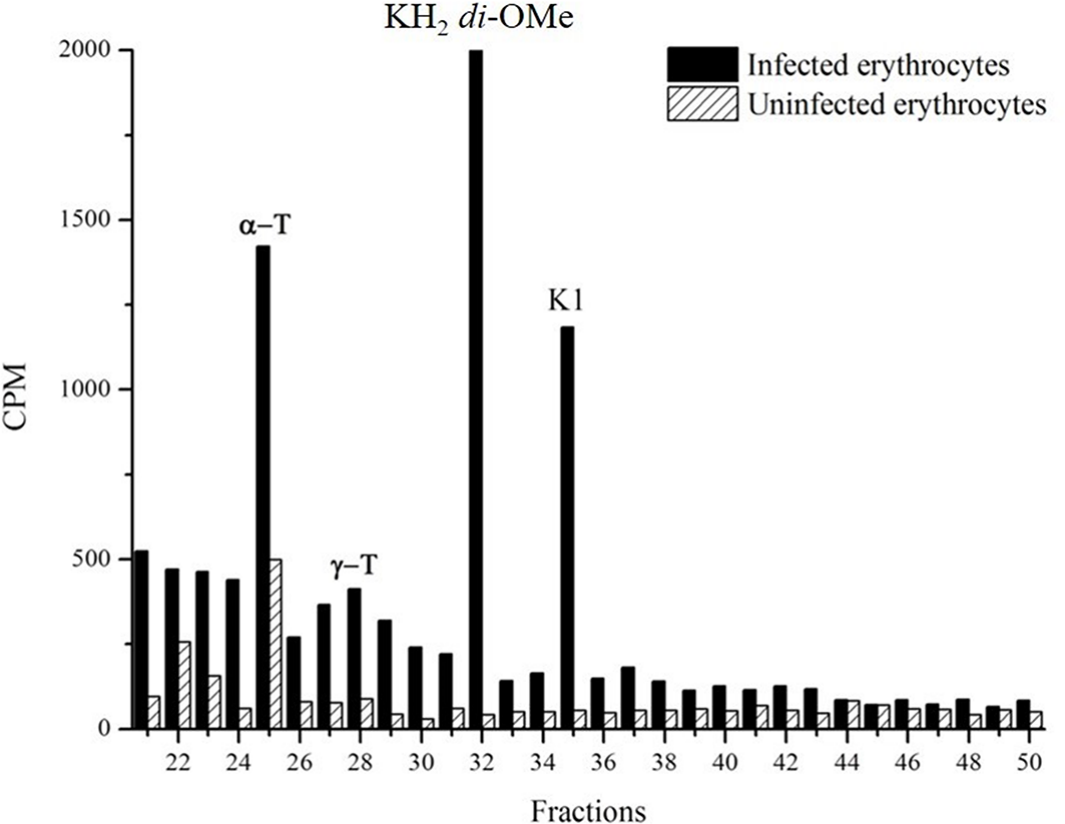
Detection of methylated hydrophylloquinone. The hydrophylloquinone were stabilized by adding methyl iodide in infected erythrocytes extract labeled whit [^3^H]-phytyl. After the extraction with dichloromethane, samples were analyzed by RP-HPLC (system I). Demonstration of the presence of radioactive fractions coincident with the standards α-T(α-tocopherol); γ-T (γ-tocopherol); KH_2_ di-*O*-Me (methylated hydrophylloquinone) and K1 (phylloquinone). The experiment was performed five times with similar results.

To determine whether probable PK *P. falciparum* can be found in the reduced form, schizonts stages were metabolically labeled with [^3^H]-phytyl-PP. The di-*O*-methylation of hydrophylloquinone was performed as previously described to stabilize the compound’s redox state (Sussmann et al., 2016). The presence of di-*O*-methylated hydrophylloquinone was confirmed by RP-HPLC using system I. The identity of derivatized hydrophylloquinone was confirmed by gas chromatography/mass spectrometry (GC/MS) analysis as described (Sussmann et al., 2016). The radioactive fractions of the parasites coincide with the α-tocopherol, methylated hydrophylloquinone and PK standards, but were not detected in uninfected blood, demonstrating that the phytylated molecule is not biosynthesized by erythrocytes (**Figure 4**).

### Presence of *Phytyl-PP* Intermediate

**Figure 5:**
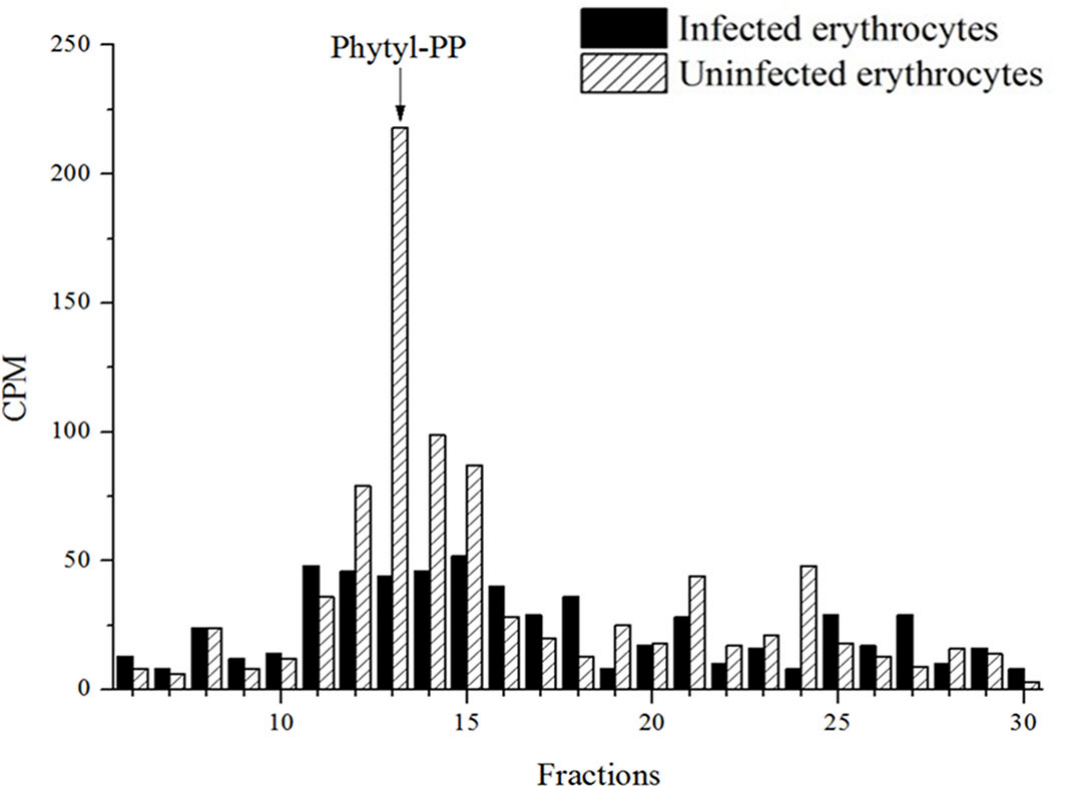
[^3^H]-GGPP radioactive incorporation profile for *P. falciparum* and uninfected erythrocytes. Infected erythrocytes with mature forms and uninfected erythrocytes metabolically labeled with [^3^H]-GGPP were subjected to extraction with n-butyl alcohol saturated with water as described for phytyl-PP extraction. The extracts were purified by RP-HPLC (system II). The experiment was performed twice with similar results.

Once the metabolite, probable PK, was characterized in both redox states in the parasite *P. falciparum*, we further investigate the biosynthesis of the isoprenoid quinone side chain. For this purpose, parasites were labeled with [^3^H]-GGPP as described. Extracts were obtained using the phytyl-PP extraction protocol and analyzed by RP-HPLC system II. The metabolic profile obtained showed radioactive incorporation in fractions containing a phytyl-PP co-injected standard (**Figure 5**) indicating the formation of phytyl-PP having GGPP as a precursor.

**Figure 6:**
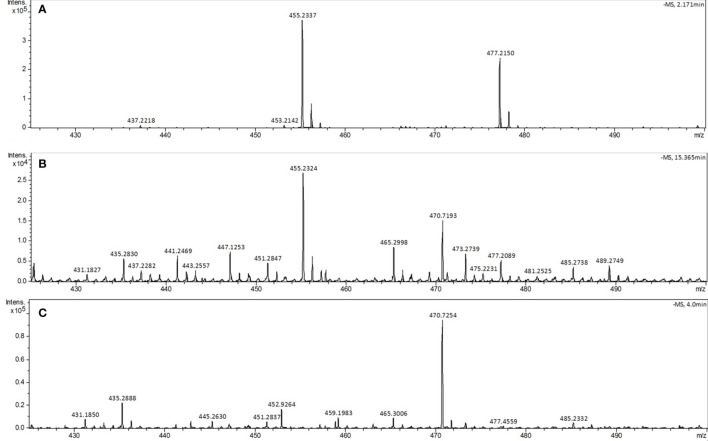
Analysis of phytyl-PP by LC-MS system II. The phytyl-PP spectra were obtained from TLC bands recovered from the samples with the same *R_f_* as the phytyl-PP standard. **(A)** Phytyl-PP standard, **(B)** infected erythrocytes and **(C)** uninfected erythrocytes. Ions: [M] - *m/z* 455.2337 and [M + Na] - *m/z* 477.2150. The experiment was performed twice with similar results.

Identification of phytyl-PP in the parasite was also performed by mass spectrometry (HRESIMS). For this purpose, 5x10^9^ infected and uninfected cells and the corresponding amount (50 mL) of lyophilized complete culture medium were used. The crude extract was submitted to thin-layer chromatography (TLC) purification as described (Ischebeck et al., 2006), and the bands corresponding to phytyl-PP were scraped, extracted with saturated butanol (1:1 v/v) and analyzed by direct injection into the LC/MS using system II (**Figure 6**). The phytyl-PP was detected in infected erythrocytes based on the ion at *m/z* 455.2324 Da corresponding to [M-H]^-^ with an error of 2 ppm comparing to calculated formulae for C_20_H_42_P_2_O_7_ (calc. *m/z* 455.2333 Da) and with the same isotopic profile as the standard. The sodium adduct [M+Na-2H]^-^ was also detected.

### Phytol Phosphorylation

**Figure 7:**
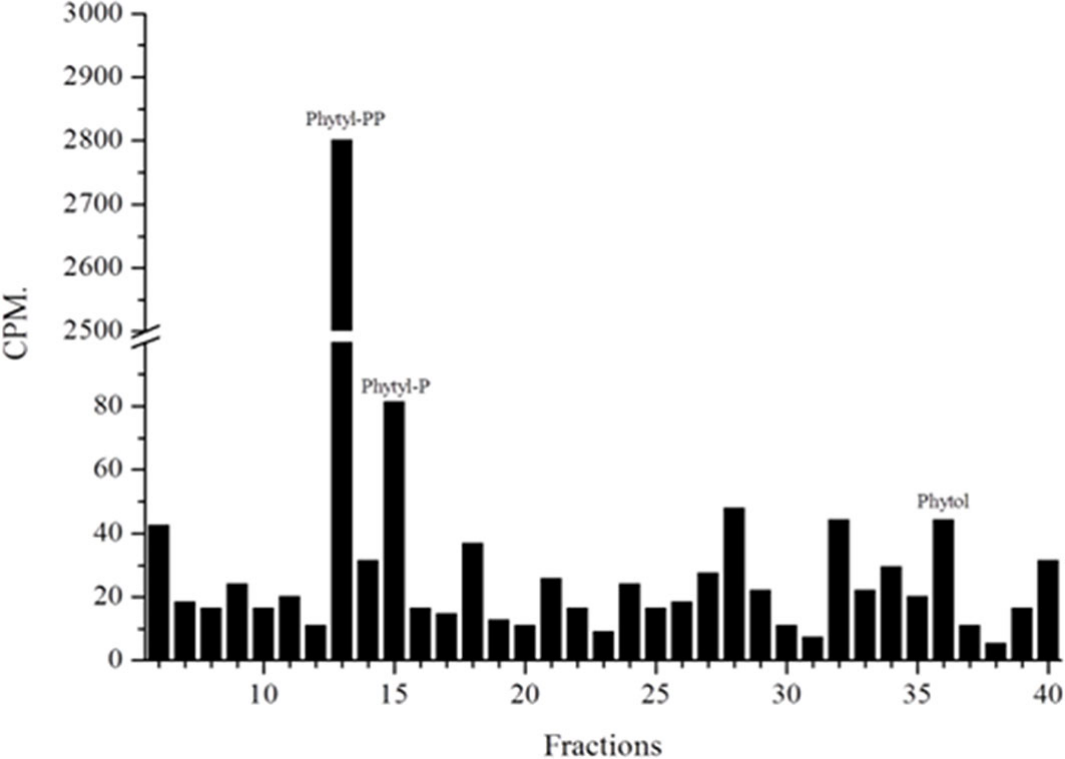
Phytol phosphorylation assay. Parasitic protein extracts labeled with [1-(n)-^3^H]-phytol were prepared as described and studied by Ischebeck et al. (9) and Valentin et al. (35) and analyzed by RP-HPLC (system II). Extracts rich in parasitic membranes were used. Fractions containing phytyl-P, phytyl-PP and phytol are indicated.

Another question we faced was whether parasites could also produce phytyl-PP *via* phytol phosphorylation. For this purpose, we followed the methods of Ischebeck et al. and Valentin et al. in previous studies (Ischebeck et al., 2006; Valentin et al., 2006). The plasmodial phosphorylation of phytol was detected (**Figure 7**) as described for *Arabidopsis* (Ischebeck et al., 2006; Valentin et al., 2006). Phytol kinase activity requires phosphate donors such as ATP, CTP, UTP, or GTP. In most organisms, various isoprenoid phosphorylation reactions are mediated by CTP (Heller et al., 1992; Inoue et al., 1994; Bentinger et al., 2007).

### Phylloquinone Attenuates Atovaquone Effects on Parasitic Growth and Respiration Rate

**Figure 8:**
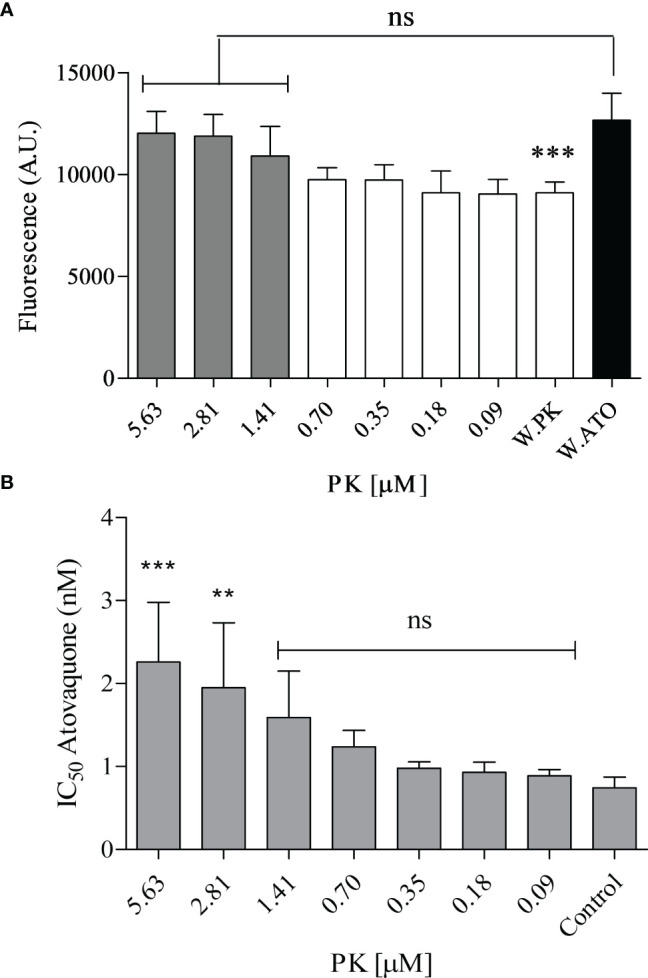
Phylloquinone effects on atovaquone-treated parasites. **(A)** Parasites were treated or not (without atovaquone; W. ATO) for 48 h with 0.6 nM ATO. At the same time, PK was applied or not (without phylloquinone; W. PK) at different concentrations ranging from 0.09 to 5.63 µM. **(B)** The IC_50_ value of ATO for parasitic growth was estimated at different concentrations of PK. The increase in fluorescence means a low inhibition of the parasite growth. Control: the IC_50_ of ATO without PK. Parasite growth was estimated by the DNA staining protocol described in section 2.14. One-way ANOVA followed by Dunnett’s posttest was used for statistical analysis. ***, *p* < 0.001; **, *p* < 0.01; ns, difference not statistically significant. All experiments were performed four times.

The antiplasmodial effect of 0.6 nM atovaquone {ATO, *trans*-2-[4-(4-chlorophenyl} cyclohexyl]-3-hydroxy-1,4-naphthalenedione) was investigated (**Figure 8A**). After treatment with different concentrations of PK, the parasite proliferation was recovered. After treatment with 1.41 µM PK, the effect of ATO was almost nullified, with growing rates similar to the control (untreated parasites). With this result, it was decided to expand the experiment by estimating the ATO IC_50_ value at different PK concentrations. The interference of PK in the antimalarial effect of ATO was confirmed by treatment with 2.81 and 5.63 µM PK, which significantly increased the ATO IC_50_ value by 2.6- and 3-fold, respectively (**Figure 8B**). It is important to note that PK by itself and at the concentrations used in our experiments does not affect parasite proliferation or the oxygen concentrations in the oxygraph (data not shown).

Since a supposed role of MK-4 in the electron transport chain had already been indicated (Tonhosolo et al., 2010), we also tried to perform tests to recover the effect of ATO with this metabolite. However, we found that when administered exogenously, MK-4 had an antimalarial effect, with an IC_50_ of 1.18 ± 0.23 µM (**Figure S3A**). At 0.25 µM concentration, MK-4 had an antiplasmodial effect; therefore, we used concentrations of 20 and 50 nM for recovery tests. As seen in **Figure S3B**, MK-4 does not recover the antiplasmodial effect of ATO at the tested concentrations. Since PK attenuated ATO, oxygen consumption assays were used to investigate whether this vitamin could directly interfere with the mechanism of action of ATO *in vitro*.

**Figure 9:**
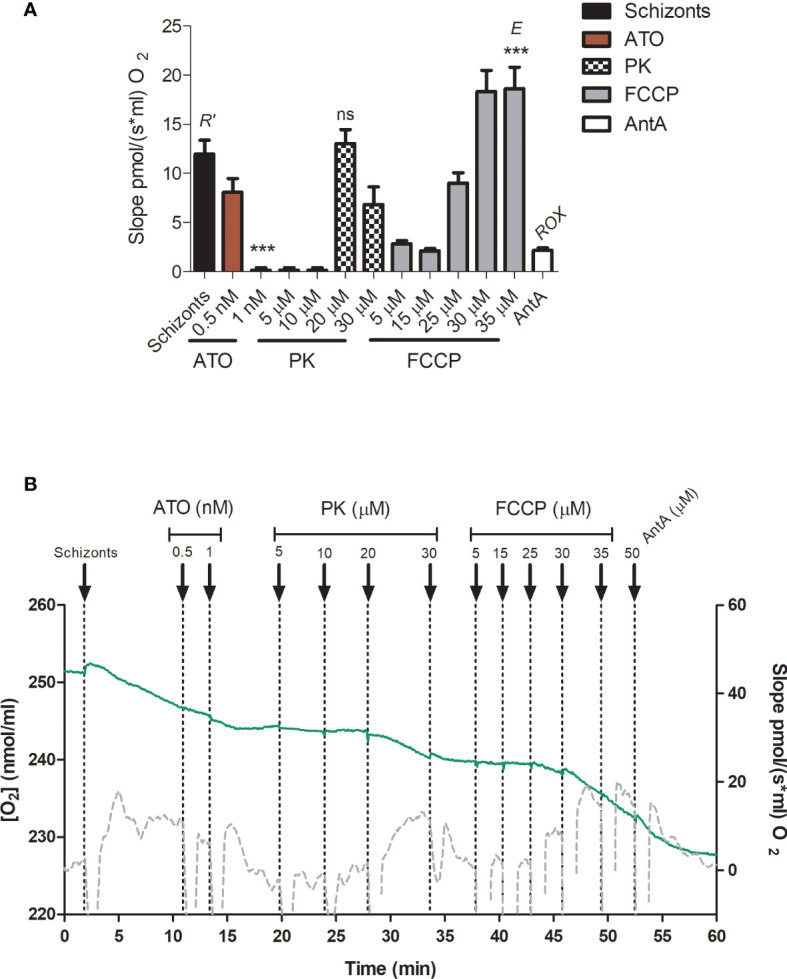
Phylloquinone effect on respiration. Oxygen consumption was measured in erythrocyte-free schizonts. **(A)** The respiration slopes obtained under particular treatments are shown. *R’*, total cellular oxygen consumption. ATO, atovaquone 0.5 and 1 nM. PK: phylloquinone 5, 10, 20 and 30 µM. FCCP: carbonyl cyanide p-trifluoro-methoxyphenyl hydrazine (5, 15, 25, 30 and 35 µM. *E*, ET capacity. AntA, Antimycin A (50 µM). ROX, residual oxygen consumption, not related to *R’*. **(B)** Green lines represent the O_2_ concentration in an oxygraph chamber as a function of time (left axis). The gray lines indicate the slope variation of oxygen concentration (right axis). As a positive control, the schizonts were maintained in respiratory buffer. One-way ANOVA followed by Dunnett’s posttest was used for statistical analysis to compare the values to R’. ***, *p* < 0.001; ns, difference not statistically significant. All experiments were performed three times.

The rates of O_2_ consumption were measured after the addition of erythrocyte-free schizonts and gave the total cellular oxygen consumption rate (*R’*) (**Figure 9A**). The parasites were treated with ATO (up to a final concentration of 1 nM). ATO is an inhibitor of mitochondrial ETS complex III and therefore, as expected, reduced the oxygen consumption. Afterward, PK was titrated up to 30 µM. Our results demonstrate that PK triggered an oxygen consumption rate similar to *R’* at 20 µM and started to decrease the rate at above 30 µM (**Figure 9B**). An additional titration with carbonyl cyanide-4-(trifluoromethoxy) phenylhydrazone (FCCP) was performed to obtain the optimum concentration of the protonophore that triggers the maximum oxygen flux/electron transfer through the ETS (*E/*ET capacity). It was verified that at 30-35 µM FCCP, the oxygen consumption slope reached *E* (**Figure 9B**), ensuring mitochondrial integrity and proper functioning of all ETS components. Furthermore, after treatment with 50 µM antimycin A (AntA), was possible to estimate that approximately 18% of *R’* corresponded to residual oxygen consumption (ROX).

## Discussion

The first report of a prenylquinone in *Plasmodium* was made by Skelton et al. (Skelton et al., 1970), who detected UQ homologs in *Plasmodium knowlesi*, *P. cynomolgi* and *P. berghei*. However, they were not able to detect vitamin K in these parasites (Skelton et al., 1970). Only 40 years later the structure and biosynthesis of vitamin K2 (MK-4) were suggested in *P. falciparum* (Tonhosolo et al., 2010). Afterwards, the detection of tocopherol in *P. falciparum* by Sussmann et al. (Sussmann et al., 2011) indicated a source for phytyl-PP to the parasite, since it is the isoprene precursor for vitamin E. Therefore, our findings indicate two hypotheses for phylloquinone fate in the parasite: (i) *P. falciparum* possess an active phylloquinone biosynthesis pathway or (ii) *P. falciparum* incorporate phylloquinone from erythrocytes and/or human plasma.

Importantly, *P. falciparum* is not the first organism that could produce different vitamin K homologs with unsaturated and saturated isoprene side chains. Full and partial reduction of the double bonds in the isoprene side chains of several menaquinones (MQs) was previously demonstrated in bacteria and archaea, including *Mycobacterium tuberculosis* (Scholes and King, 1965; Tindall, 1989; Hemmi et al., 2005; Upadhyay et al., 2015). In the parasite, radiolabeled phytyl/phytol incorporation into PK indicates phytylation, but the possibility of enzymatically reducing the MQ-4 side chain cannot be ruled out.

Metabolic labeling with [^3^H]-GGPP or [^3^H]-FPP and their incorporation into tocopherols and PK suggests that the parasite would be able to *de novo* biosynthesize the PK side chain. Moreover, incorporation with *S*-adenosyl-*L*-[methyl-^3^H] methionine in peaks coinciding with those of the PK suggests the presence of SAM-mediated methylations through PK biosynthesis, which is required for most prenylquinone biosynthesis (Threlfall et al., 1968; Nowicka and Kruk, 2010; Basset et al., 2017; Verdaguer et al., 2019). In addition, when cultures of *P. falciparum* in its intraerythrocytic stages were labeled with [^3^H]-phytyl, peaks coinciding with those of the PK and tocopherol standards were detected.

The presence of PK in the parasite was confirmed by high-resolution mass spectrometry using chromatographic separation and atmospheric pressure chemical ionization LC-APCI-QToF. The chromatographic signal and the intensity of the fragments and related ions were above the detection limit, allowing unequivocal confirmation of phylloquinone in infected erythrocytes.

The PK in the parasite was also characterized by electrochemistry techniques. Electrochemistry experiments were performed on films of the vitamin K1 standard, uninfected erythrocytes and *P. falciparum*-infected erythrocytes in contact with PBS. Erythrocytes displayed well-defined signals for the Fe(III)-heme/Fe(II)-heme redox couple, whereas vitamin K1 produced signals associated with the interconversion between its oxidized and reduced forms, involving the two-proton, two-electron transfer associated with quinone/catechol motifs. Analysis of such signals confirmed the presence of vitamin K1 in the erythrocytes infected with *P. falciparum*, some of which (5-10%) was in its reduced form. The presence of reduced PK was also confirmed by stabilizing the redox state of prenylquinone through methylations and then detecting it by metabolic labeling techniques (Sussmann et al., 2016).

The most studied function of PK is related to electron transport in photosystem I, but other functions have been suggested, such as oxidative folding of photosystem II subunits (Nowicka and Kruk, 2010); however, this is not the only PK function in plants. For example, PK can also participate in protection from ROS under stress conditions and against pathogen attacks in plants (Collins et al., 1977; Soballe and Poole, 1999; Lochner et al., 2003; Nowicka and Kruk, 2010; Sogi et al., 2016). Furthermore, some studies indicate that vitamin K, in its reduced form (hydroquinone), acts as an antioxidant in the lipid phase of cells (Fiorentini et al., 1997; Vervoort et al., 1997). Recently, it was demonstrated an alternative targeting of phylloquinone for transmembrane redox signaling associated with parasitism in nonphotosynthetic holoparasites plants (Xi et al., 2021). The presence of PK in plant membranes suggests the involvement of PK-mediated ETS in plant plasma membranes acting as a redox mediator (Lochner et al., 2003). Considering that *P. falciparum* is not a photosynthetic organism, PK should participate in other cellular redox processes.

Therefore, we investigated the effects of PK on mitochondrial ETS. We tested using PK to replace UQ in its classically described function in mitochondrial ETS. It is important to note that ATO is considered an analog of UQ, but the molecule is structurally more related to PK.

ATO is an antimalarial agent (Hudson, 1993) whose mechanism of action is believed to specifically be competing with ubiquinol (UQH_2_) to interact with the mitochondrial bc_1_ complex to prevent protozoa from redox-recycling UQ, which is required for dihydroorotate dehydrogenase (DHODH) activity (Painter et al., 2007). Importantly, ATO toxicity is extremely decreased in recombinant parasites because of a DHODH enzyme is not dependent on UQ (Painter et al., 2007), which suggests that the major lethal effects of the compound are caused by the lack of UQ redox regeneration required for pyrimidine biosynthesis. Therefore, soluble UQ analogs can probably rescue the antimalarial effects on parasitic growth by replacing ATO at the mitochondrial bc_1_ complex or directly being used by DHODH (Canfield et al., 1995). Similarly, we examined how exogenous addition of 20 μM vitamin K1 can attenuate ATO effects on parasitic growth. In contrast, vitamin K2 does not possess this capacity, at least at the nontoxic concentrations tested here on the nanomolar scale (**Figure S3**). Moreover, PK also effectively restored the parasitic oxygen uptake inhibited by ATO.

All the above-mentioned results suggest that PK can functionally act as an electron carrier for at least DHODH and mitochondrial complex III. In addition to DHODH research, further study is needed to determine whether other cellular dehydrogenases/oxidases can functionally use PK for their activity. For now, it was demonstrated only that mitochondrial NADH dehydrogenase from *P. falciparum* can bind to both MK-4 and menadione *in vitro* (Dong et al., 2009; Tonhosolo et al., 2010). It is important to note that all results presented here were obtained by the addition of exogenous PK; thus, it is unknown whether the prenylquinone involvement in the respiratory process occurs naturally and is involved in other cellular processes. Since ATO was demonstrated to be an excellent antimalarial agent, the biosynthesis of respiratory quinones may be related to its efficacy and resistance phenomena. Therefore, PK and/or UQ biosynthesis inhibitors could be effective drugs for malaria. Moreover, vitamin K1 studies could lead to the development of parasite-specific drugs since their biosynthesis and potential mitochondrial function did not occur in humans (Nowicka and Kruk, 2010; Verdaguer et al., 2019).

In addition to PK eventual involvement in mitochondrial processes, two other functions for prenylquinone in the apicomplexan parasite can be suggested. First, it has been speculated that hydrophylloquinone (**Figure 4**) can participate in the adhesion of infected erythrocytes to capillaries due to the associated coagulation mechanism (Basset et al., 2017). Therefore, the possibility of the parasite biosynthesizing vitamin K1 to promote this process cannot be ruled out. The second possibility is that vitamin K1 could be involved in plasma membrane redox systems, as was described for other similar naphthoquinones identified in *Leishmania donovani* (chlorobiumquinone) and *Entamoeba histolytica* (thermoplasmaquinone) (Fiorentini et al., 1997; Vervoort et al., 1997; Biswas et al., 2008; Nandi et al., 2011). Similar to vitamin K biosynthesis in *Plasmodium*, the formation of chlorobiumquinone and thermoplasmaquinone in protozoa has not been elucidated. In fact, bioinformatics approaches do not reveal sequences involved in vitamin K biosynthesis in the *L. donovani* and *E. histolytica* genomes (based on information from the EuPathDB database, https://eupathdb.org/eupathdb/. Last access: January 2022).

Phylloquinone and tocopherols have different aromatic rings, a chromanol group for tocopherol and a 1,4-naphthoquinone in the case of PK. However, the isoprene side chain derived from phytyl-PP is the same for both prenylquinones. Metabolic techniques were used to demonstrate the incorporation of phytyl-PP, phytol and GGPP into PK. These results are indirect evidence that phytol/phytyl-PP biosynthesis and phytol phosphorylation pathways must be active in the parasite for PK biosynthesis. Therefore, we attempted to directly observe phytol phosphorylation by the parasite, as it occurs in other organisms (Ischebeck et al., 2006; Valentin et al., 2006). A chromatographic study of schizont extracts showed that phytol kinase activity was especially high in parasitic membranes.

Finally, since the parasite supposedly has no chlorophyll, phytol/phytyl-PP are probably obtained through the direct incorporation from extracellular medium or *via* hydrogenation of GGPP/geranylgeraniol, catalyzed by GGRs (Sasaki et al., 2011; Meadows et al., 2018). GGRs are NADPH- or FADH-dependent enzymes already characterized in archaea, algae, plants, cyanobacteria and bacteria (Sasaki et al., 2011; Meadows et al., 2018). Additionally, several *P. falciparum* strains possess a putative polyprenol reductase in their genomic database (PF3D7_1455900 in *P. falciparum* 3D7; based on information from the PlasmoDB database, https://plasmodb.org/plasmo/app/record/gene/PF3D7_1455900. Last access: January 2022). Further studies will be required to better understand the transport of phytol/phytyl-PP and/or the enzymatic hydrogenation of GGPP/geranylgeraniol in the parasite.

Here, we report the first proposal of PK mitochondrial function and the first evidence of PK in the malaria parasite. PK and phytol/phytyl-PP biosynthesis together with the phytol salvage pathway are typical pathways in photosynthetic organisms. Their presence in the parasite could be another consequence of *Plasmodium* evolutionary history, and their study allows us to understand parasite biology. Both pathways are absent from humans, so further confirmatory experiments involving the PK biosynthesis pathway, its biological role, and phytyl-PP metabolism as potential drug targets against malaria is suggested.

## Data Availability Statement

The original contributions presented in the study are included in the article/**Supplementary Material**. Further inquiries can be directed to the corresponding author.

## Author Contributions

RS and AK designed the study. Experiments were performed by RS, HG, AR, DM, LY, AD-C, GC-T, IB, and MC. RS, HG, AR, DM, LY, AD-C, GC-T, EK, MK, and AK analyzed the data and contributed to interpretation of the data. The first draft of the manuscript was written by RS. All authors commented and contributed to the article and approved the final version of the manuscript.

## Funding

This research was supported by grants from the FAPESP (project 2017/2245-1 AK and 2014/50316-7 MK), CAPES and the CNPq.

## Conflict of Interest

The authors declare that the research was conducted in the absence of any commercial or financial relationships that could be construed as a potential conflict of interest.

## Publisher’s Note

All claims expressed in this article are solely those of the authors and do not necessarily represent those of their affiliated organizations, or those of the publisher, the editors and the reviewers. Any product that may be evaluated in this article, or claim that may be made by its manufacturer, is not guaranteed or endorsed by the publisher.
