# Supplementary material for: Presence of Phylloquinone in the Intraerythrocytic Stages of Plasmodium falciparum
Source: Front Cell Infect Microbiol. 2022 Apr 21;12:869085. doi: 10.3389/fcimb.2022.869085 (PMC9069557; doi:10.3389/fcimb.2022.869085)
Supplement: Supplementary file 1 [file DataSheet_1.docx]

**Supplementary Material**

**
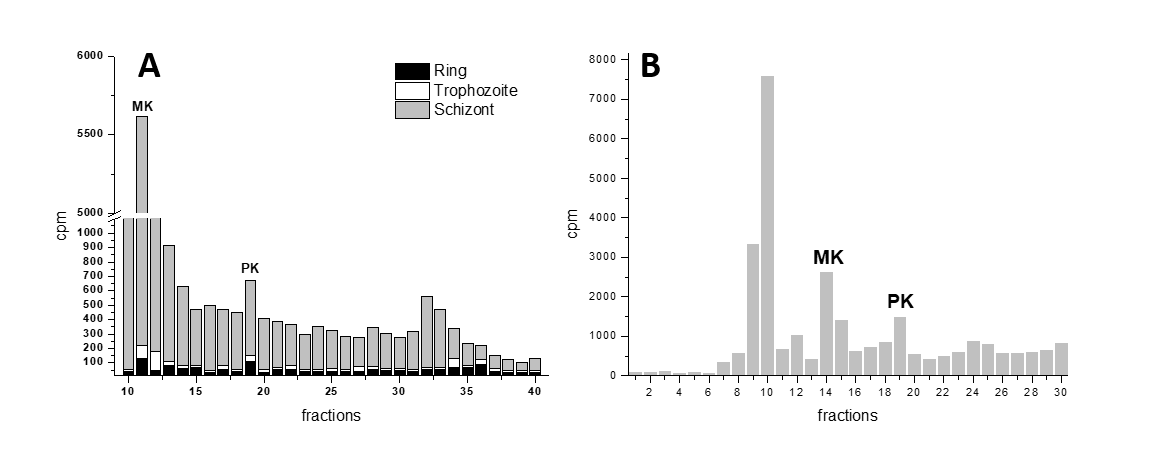
**

**Figure S1. Radioactive quinone incorporation profile for *P. falciparum****.* Erythrocytes infected with parasites in three different stages metabolically labeled with [^3^H]-GGPP (A) or in mature stages metabolically labeled with [^3^H]-FPP (B) were subjected to extraction. The extracts were separated by RP-HPLC (system III). Radioactive fractions were coincident with the corresponding standards MK (menaquinone) and PK (phylloquinone).


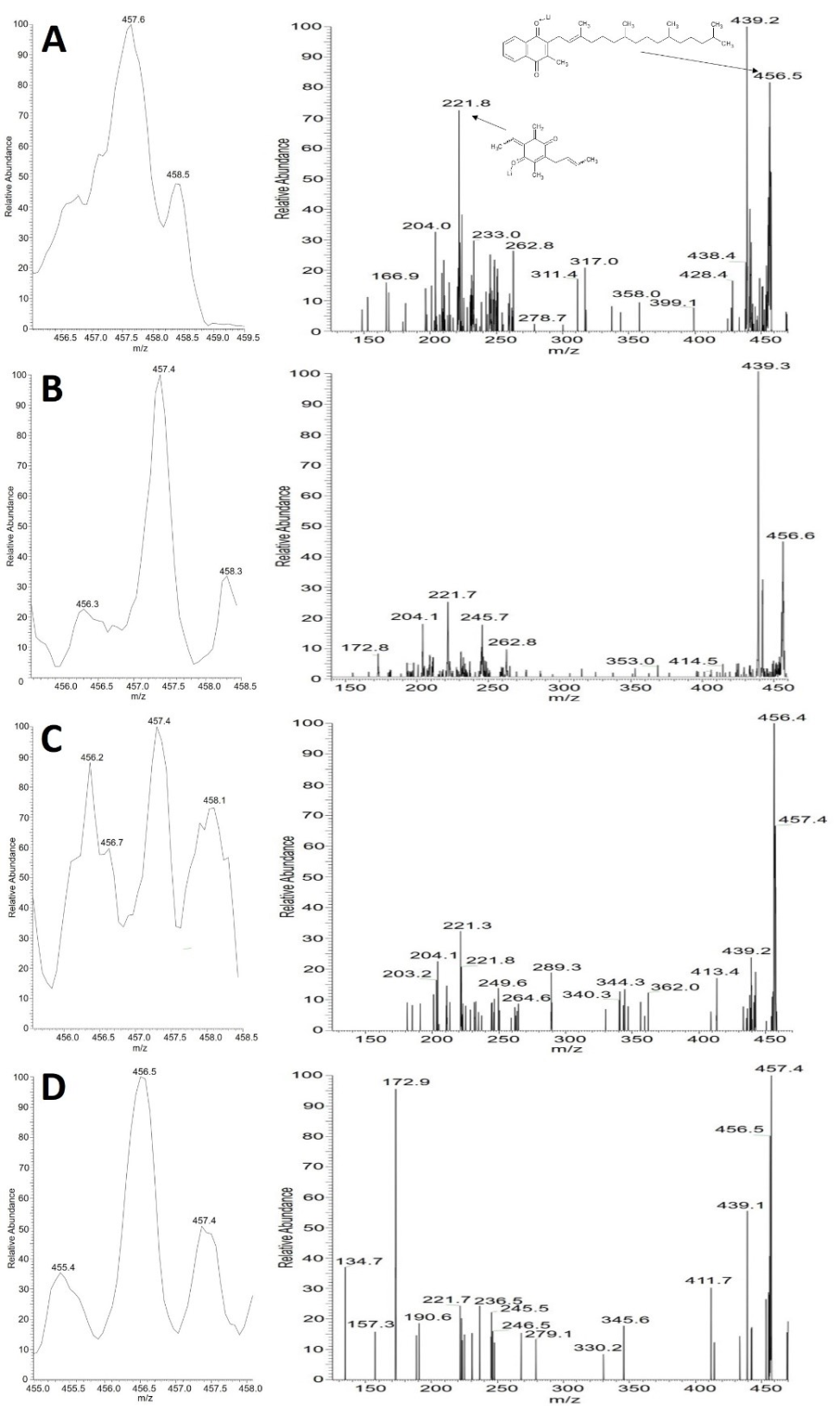


**Figure S2. Detection of phylloquinone by mass spectrometry system I.** The fractions purified from RP-HPLC system I corresponding to the retention time of vitamin K1 from three different extracts were analyzed. Vitamin K1 standard (A); Infected erythrocytes (B); Uninfected erythrocytes (C); Culture medium (D). The spectra obtained in the samples were compared with that of standard PK. Chemical structures of the molecular ion (left) and the most important breakdown products are shown (right). Experiment was performed three times with similar results.


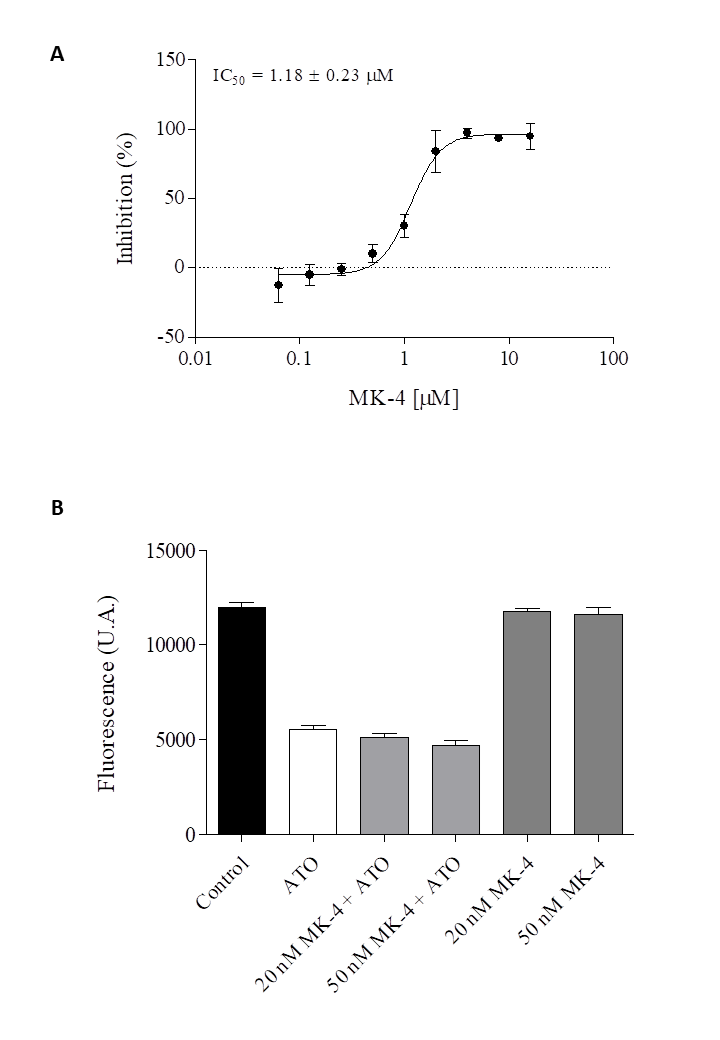


**Figure S3. MK-4 toxicity for *P. falciparum* and efforts to recover the ATO effect***.* (A) The IC_50_ value of MK-4 in parasitic growth was estimated using different concentrations of MK-4: 16 µM was the first concentration of ATO, which reached 0.0625 µM by serial dilution (1:1/vol:vol). (B) Infected cultures were treated or not (Control) for 48 h with 0.6 nM atovaquone (ATO). At the same time, MK-4 was applied at two different concentrations, 20 or 50 µM, to ATO-treated or untreated cultures.
